# Quantitative proteomic characterization of lung tissue in idiopathic pulmonary fibrosis

**DOI:** 10.1186/s12014-019-9226-4

**Published:** 2019-02-06

**Authors:** Yaqiong Tian, Hui Li, Yujuan Gao, Chuanmei Liu, Ting Qiu, Hongyan Wu, Mengshu Cao, Yingwei Zhang, Hui Ding, Jingyu Chen, Hourong Cai

**Affiliations:** 1 Department of Respiratory Medicine, The Affiliated Drum Tower Hospital of Nanjing University Medical School, No. 321 Zhongshan Road, Nanjing, 210008 Jiangsu People’s Republic of China; 2 Department of Respiratory Medicine, Yi Ji Shan Hospital of Wannan Medical College, No. 2 Zheshan West Road, Wuhu, 241001 Anhui People’s Republic of China; 3 Department of Respiratory Medicine, KunShan Hospital of Traditional Chinese Medicine, No. 189 Chaoyang Road, Kunshan, 215300 Jiangsu People’s Republic of China; 4 Department of Pathology, The Affiliated Drum Tower Hospital of Nanjing University Medical School, No. 321 Zhongshan Road, Nanjing, 210008 Jiangsu People’s Republic of China; 5 Department of Respiratory Medicine, Yixing People Hospital, Affiliated Jiangsu University, No. 75 Tongzhenguan Road, Yixing, 214200 Jiangsu People’s Republic of China; 6 Jiangsu Key Laboratory of Organ Transplantation, Wuxi People’s Hospital, Nanjing Medical University, No. 299 Qingyang Road, Wuxi, 214023 Jiangsu People’s Republic of China

**Keywords:** Idiopathic pulmonary fibrosis, Proteomic, Extracellular matrix, Isobaric tag for relative and absolute quantitation (iTRAQ)

## Abstract

**Background:**

Idiopathic pulmonary fibrosis (IPF) is a progressive, eventually fatal disease. IPF is characterized by excessive accumulation of the extracellular matrix (ECM) in the alveolar parenchyma and progressive lung scarring. The pathogenesis of IPF and whether the ECM involved in the process remain unknown.

**Methods:**

To identify potential treatment target and ECM associated proteins that may be involved in the development of IPF, we employed isobaric tag for relative and absolute quantitation (iTRAQ) combined liquid chromatography–tandem mass spectrometry (LC–MS/MS) approach to examine protein expression in lung tissues from IPF patients.

**Results:**

A total of 662 proteins with altered expression (455 upregulated proteins and 207 downregulated proteins) were identified in lung tissue of IPF patients compared with control. KEGG pathway enrichment analysis showed that the altered proteins in lung tissue mainly belonged to the PI3K-Akt signaling, focal adhesion, ECM-receptor interaction, and carbon metabolism pathways. According to the bioinformatic definition of the matrisome, 229 matrisome proteins were identified in lung tissue. These proteins comprised the ECM of lung, of which 104 were core matrisome proteins, and 125 were matrisome-associated proteins. Of the 229 ECM quantified proteins, 56 significantly differentially expressed proteins (19 upregulated proteins and 37 downregulated proteins) were detected in IPF lung tissue samples. In addition to proteins with well-known functions such as COL1A1, SCGB1A1, TAGLN, PSEN2, TSPAN1, CTSB, AGR2, CSPG2, and SERPINB3, we identified several novel ECM proteins with unknown function deposited in IPF lung tissue including LGALS7, ASPN, HSP90AA1 and HSP90AB1. Some of these differentially expressed proteins were further verified using Western blot analysis and immunohistochemical staining.

**Conclusions:**

This study provides a list of proteomes that were detected in IPF lung tissue by iTRAQ technology combined with LC–MS/MS. The findings of this study will contribute better understanding to the pathogenesis of IPF and facilitate the development of therapeutic targets.

**Electronic supplementary material:**

The online version of this article (10.1186/s12014-019-9226-4) contains supplementary material, which is available to authorized users.

## Background

Idiopathic pulmonary fibrosis (IPF) is a progressive disease that is the most common and lethal type of idiopathic interstitial pneumonia. IPF is characterized by scarring fibrosis with a median survival of 2–3 years after diagnosis and has an unpredictable progression [1–3]. This high mortality rate and relatively few therapeutic options are partly due to an incomplete understanding of the molecular mechanisms behind the disease [4, 5]. High-throughput biotechnologies have enabled the collection of unprecedented numbers of omics datasets to uncover underlying mechanisms related to the genesis and development of various diseases [6, 7]. Moreover, recent key advances in transcriptomic studies have elucidated new potential mechanisms and therapeutic targets and have also advanced the role of “omics” in IPF [6–8]. For instance, MUC5B polymorphism was found to increase the disease susceptibility of IPF. CCNA2 and alpha-defensin genes were upregulated in IPF lung tissues with acute exacerbations [9–11]. CD28, ICOS, LCK, and ITK in peripheral blood mononuclear cells (PBMCs) were discovered to be predictors of poor outcomes in IPF by omics approaches [12–15]. Recent study have focused on metabolic changes in bleomycin-treated mouse lung and IPF patient lung, proteomic analysis was performed on these lung tissues. They observed reduced glycolysis/gluconeogenesis and enhanced ascorbate and aldarate metabolism, and the imbalanced metabolism can be restored by pirfenidone [16]. Proteomic analysis also have been applied to identify serum biomarkers for IPF, they found that 97 out of total 394 proteins were associated with IPF, C-reactive protein (CRP), fibrinogen- α chain, haptoglobin, and kininogen-1 were useful candidate biomarkers for IPF [17]. Another proteome analysis for serum of IPF patients also found that CRP is a biomarker for the diagnosis of IPF [18]. Different forms of organ fibrosis may share common protein regulation, identifying common and specific targets can promote the treatment for tissue fibrosis. For example, proteome analysis for human lung and skin fibrosis reveals surprisingly high prevalence of marginal zone B- and B1-cell-specific protein (MZB1)-positive plasma B cells both in lung and skin fibrosis [19].

These studies suggested that the use of omics analysis is a strategic mean to unearth the molecular mechanisms, diagnostic biomarkers and new therapeutic targets of the disease. And integrated analysis to multi-omics datasets derived from genomics, transcriptomics, epigenomics, proteomics, microbiome and metabolomics allow us connect all the seemingly unrelated pieces of information to build a mechanism model for IPF [20]. However, compared with other omics analysis, proteomics data for IPF lung tissue is relatively limited so far.

The etiology and pathogenesis of IPF remain incompletely understood. The pathophysiology of IPF features a paradigm that involves injury, loss of the epithelial cell barrier with aberrant re-epithelialization, fibroblast activation and unregulated deposition of extracellular matrix (ECM) components in myofibroblasts [4, 5]. Excessive accumulation of ECM in the alveolar parenchyma and progressive scarring of lung tissue are major characteristics of IPF [21]. ECM components influence myofibroblast differentiation by modulating fibrogenic growth factor activity (e.g. TGF-β) and mechanotransductive pathways, which in turn alter ECM mechanical properties [22]. An understanding of the synthesis and degradation of ECM components in IPF plays a key role in identifying novel treatment targets for this chronic debilitating disease. However, to the best of our knowledge, limited proteomics studies have focused on IPF lung tissues [23–27]. Several studies have elaborated ECM compositions in IPF by studying biopsy samples, resected tissue or autopsy samples taken from the lungs [27]. Recently study has detected ECM deposition properties in IPF patient- derived fibroblasts/myofibroblasts by mRNA, miRNA, and proteomic analysis. They found that 227 matrisome were detected in at least one sample, and ECM changes were the most predominant feature in their results [28].

In this study, we applied a coupled iTRAQ/LC–MS/MS-based technology to identify on a large number of differentially expressed proteins in the lung tissue of IPF patients compared with controls. We used this information to describe the biological processes and molecular pathways involved in IPF pathogenesis. We further analyzed the extracellular matrices in IPF lung tissues and compared them to normal lung tissue. According to the bioinformatic definition of the matrisome [29, 30], altered ECM proteins were categorized as core matrisome proteins and matrisome-associated proteins. Core matrisome proteins comprise of ECM glycoproteins, collagens and proteoglycans. Matrisome-associated proteins include ECM-affiliated proteins, ECM regulators (ECM remodeling enzymes and their regulators) and ECM-associated secreted factors. The goal of this work was to identify altered proteins and understand biological pathways variations and aberrant ECM components -in IPF. These results contribute toward discovering potential targets that interrupt IPF development and provide a basis for understanding the role of ECMs in IPF.

## Methods

### Patient description

Human lung tissue samples were obtained from 20 patients with IPF (mean age ± SD: 63.86 ± 10.58 years; 1 female, 19 males) who had undergone lung transplantation surgery in the Lung Transplant Center of Wuxi People’s Hospital (Wuxi, PR China). The diagnosis of IPF was verified by histological examination of the explanted lungs by pathologists. All patients fulfilled the diagnostic criteria for IPF as defined by the ATS/ERS/JRS/ALAT statement for idiopathic pulmonary fibrosis: evidence-based guidelines for diagnosis and management. Control lung tissues (mean age ± SD: 63.67 ± 8.27 years; 3 females, 17 males) were collected from 20 patients undergoing surgery for cancer or pulmonary nodules in the Thoracic Surgery Department of Nanjing Drum Tower Hospital. Samples were stored at − 80 °C after collection.

### Sample preparation

For the proteome analysis, frozen lung tissue samples (size 1 cm^3^) from IPF patients, and normal lung tissues were used. SDT buffer (4% SDS, 100 mM DTT, 150 mM Tris–HCl pH 8.0) was added to the samples, and the mixture was transferred to 2 ml tubes with quartz sand. The lysate was homogenized using an MP Fastprep-24 automated homogenizer (24 × 2, 6.0 M/S, 60 s, twice). The homogenate was sonicated, and then it was boiled for 15 min. After centrifuging at 14,000×*g* for 40 min, each lung homogenate was filtered with a 0.22 µm filter. The protein concentration of the filtrate was quantified using a BCA protein assay kit (P0012, Beyotime). The samples were stored at − 80 °C. To reduce biological variation from patient to patient, 4 pooled samples both in the IPF group and the control group were used. Each pooled sample contained 5 lung tissues from patients with IPF or control subjects undergoing surgery for cancer or pulmonary nodules, respectively.

### Filter-aided sample preparation (FASP digestion) and iTRAQ labeling

Prior to the iTRAQ labeling experiments, equal quantities of 200 μg proteins from each sample were incorporated into 30 μl SDT buffer. The detergent, DTT, and other low molecular weight compounds dissolved in UA buffer (8 M urea, 150 mM Tris–HCl, pH 8.5) were removed by repeated ultrafiltration (Sartorius, 30kD). Then, 100 μl iodoacetamide (100 mM IAA in UA buffer) were added to block reduced cysteine residues; the resultant solution was incubated for 30 min in darkness. Filters were washed with 100 μl UA buffer three times and then 100 μl dissolution buffer (DS buffer) twice. The protein suspensions were digested with 4 μg trypsin (Promega) in 40 μl DS buffer overnight at 37 °C. Peptides were collected by filtration. Peptide content was estimated by UV light at 280 nm spectral density using an extinction coefficient of 1.1 of 0.1% (g/l) solution that was calculated based on the frequency of tryptophan and tyrosine in vertebrate proteins.

Subsequently, 100 μg peptide mixture of each sample were performed using an 8-plex iTRAQ labeling kit (Applied Biosystems) according to manufacturer’ protocols. Four pooled control samples were labelled with iTRAQ reagents 113, 114, 115 and 116, respectively, whereas 4 pooled IPF samples were labelled with iTRAQ reagents 117, 118, 119 and 121. The concentrated iTRAQ reagent-labeled digested samples from the eight groups were solubilized in 250 μl of loading buffer A (20 mM ammonium formate, pH 10) and combined into one tube prior to fractionation.

### Peptide fractionation with reversed phase (RP) chromatography

Fractionation of iTRAQ labeled peptide mixture was performed by RP chromatography using an Agilent 1260 infinity II HPLC instrument. Dried peptide mixture was reconstituted with buffer A (10 mM HCOONH4, 5% ACN, pH 10.0) and loaded onto a XBridge Peptide BEH C18 Column, 130Å, 5 µm, 4.6 mm × 100 mm column. Peptides were eluted at a flow rate of 1 ml/min with a gradient of 0–7% buffer B (10 mM HCOONH4, 85% ACN, pH 10.0) for 5 min, 7–40% buffer B for 5-40 min, 40%–100% buffer B for 45–50 min, and 100% buffer B for 50–65 min. The elution was monitored by a UV absorbance at 214 nm/280 nm, and fractions were collected every 1 min from 5 to 50 min. Collected fractions were dried using vacuum centrifugation and separated into ten fractions.

### Mass spectrometry easy nLC

Each fraction was prepared for nano LC–MS/MS analysis. The peptide mixtures were loaded onto a C18-reversed phase analytical column (Thermo Scientific, Acclaim PepMap RSLC 50 μm × 15 cm, nano viper, P/N164943) in buffer A (0.1% formic acid) and separated with a linear gradient of buffer B (80% acetonitrile and 0.1% formic acid) at a flow rate of 300 nl/min controlled by IntelliFlow Technology with a linear gradient: 6% buffer B for 5 min, 6–38% buffer B for 45 min, 38–100% buffer B for 5 min, and hold in 100% buffer B for 5 min.

### LC–MS/MS analysis

LC–MS/MS analysis was performed on a Q Exactive mass spectrometer (Thermo Scientific) coupled with Easy nLC (Thermo Fisher Scientific) for 60 min. The mass spectrometer was operated in positive ion mode. MS data were acquired using a data-dependent top 10 method dynamically choosing the most abundant precursor ions from the survey scan (350–1800 m/z) for hot carrier diode (HCD) fragmentation. The automatic gain control (AGC) target was set to 3e6, and the maximum injection time was set at 50 ms. Survey scans were acquired at a resolution of 70,000 at m/z 200. The HCD spectra resolution spectra was set to 17,500 at m/z 200, and the isolation width was 2 m/z. Normalized collision energy was 30 eV.

### Data analysis

MS/MS spectra were searched using MASCOT engine (Matrix Science, London, UK; version 2.5) embedded into Proteome Discoverer 2.1(Thermo Fisher Scientific Inc). Protein database uniprot_HomoSapiens was used in this project. For protein identification, thresholds of 20 ppm for precursor mass tolerance and 0.1 Da for fragment mass tolerances were set. The analyses allowed for two missed cleavages from trypsin digest. Oxidation (M), acetyl (Protein N-term), and deamidated (NQ) were set as potential variable modifications; carbamidomethyl (C) was set as static modifications. The spectra against a decoy database to estimate the false-discovery rate of our identified peptides was 1%. Proteome Discoverer 2.1 software was used to determine the peak integration of peptides through the Most Confident Centroid.

### Bioinformatics analysis

Gene Ontology (GO) annotation, biological process (BP), cellular component (CC) and molecular function (MF) analyses were performed on differentially expressed proteins. The KEGG database (http://www.genome.jp/kegg/) was used to classify the identified proteins. The Search Tool for the Retrieval of Interacting Genes/Proteins (STRING) database of physical and functional interactions was used to analyze the protein–protein interaction (PPI) of selected proteins.

### Validation of differentially expressed proteins

To verify the results of the proteomes study, significantly altered proteins were selected for Western blot and immunohistochemistry in the samples, which have been used in proteomics analysis.

### Western blot

Total proteins in lung tissue were extracted following manufacturer’s instructions (Keygene, China). Protein samples were loaded on 10% SDS-PAGE gels, transferred onto PVDF membranes (Merck Millipore, Germany), blocked with 5% nonfat milk in TBST and incubated with primary antibody at room temperature for 4 h or overnight at 4 °C. The gels were subsequently incubated with HRP-conjugated secondary antibody for one hour. Protein expression was detected using ECL (Merck Millipore, Germany). Primary antibodies, i.e., COL1A1, CTSB, AGR2, SCGB1A1, LGALS7, DMBT1, MME and CAV1, were purchased from Abcam (United Kingdom), and HSP90AA1 was purchased from Enzo Life Science (United States).

### Immunohistochemistry

Fresh lung tissue was fixed by formalin and embedded in paraffin. Immunohistochemical staining for target proteins and hematoxylin–eosin staining (HE) were performed. Paraffin Sections (4 µm) were deparaffinized in xylene and rehydrated in decreasing concentrations of ethanol followed by distilled water. Endogenous peroxidase was quenched with aqueous 3% hydrogen peroxide for 15 min. Antigen retrieval was performed in a pressure cooker filled with 1 mM EDTA buffer (PH 8.0). After incubating in primary antibodies incubated overnight at 4 °C, horseradish peroxidase-conjugated secondary antibody was added for 20 min and 3,3-diaminobenzidine tetrahydrochloride (DAB) for 10 min at room temperature.

## Results

### Identification and functional ontology classification of differentially expressed proteins

**Fig. 1:**
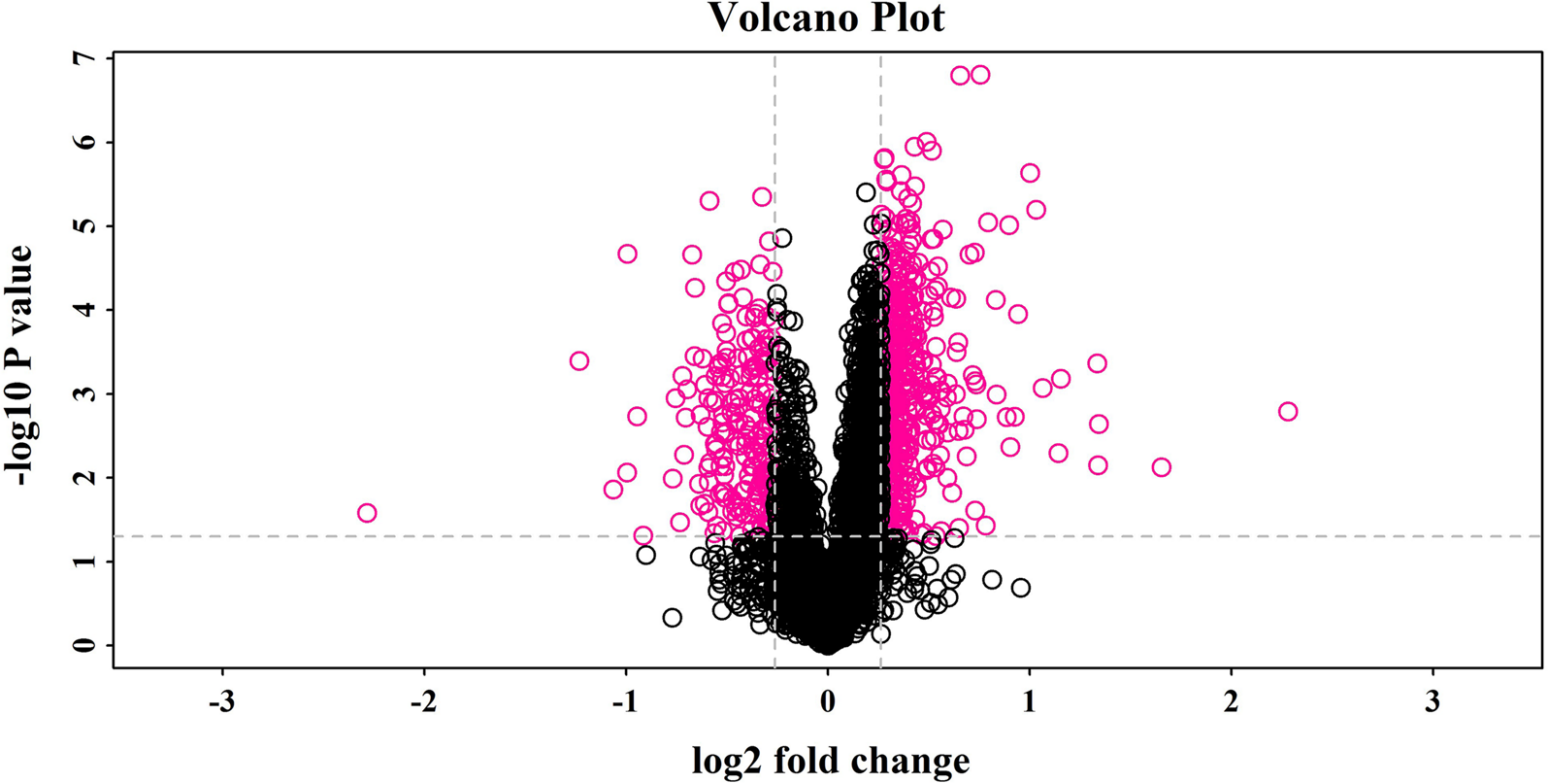
Volcano plot of differentially expressed proteins in lung tissue. This is a volcano plot of the log2 fold-change (x-axis) versus −log10 *p* value (the y-axis represents the probability that the protein was differentially abundant). The red points in the upper right (ratio > 1.2) and upper left (ratio < 0.80) sections with p < 0.05 represent proteins that were significantly dysregulated in IPF patients

A total of 4241 proteins were identified in the lung tissue samples from IPF patients and controls, matched in database (uniprot_HomoSapiens), more details were supplied in Additional file 1: Table S1. Among the 4241 identified proteins, we identified 662 significantly differentially expressed proteins (207 downregulated and 455 upregulated) in comparison between IPF patients and controls (Additional file 2: Table S2). A volcano plot based on the 662 differentially expressed proteins is shown in Fig. 1.

Among the 662 differentially expressed proteins, 229 ECM proteins were identified as matrisome proteins in normal and IPF lungs (as defined by [29, 30]), including 104 core ECM proteins (glycoproteins, collagens, and proteoglycans) and 125 ECM-related proteins (Table 1). More details can be found in Additional file 3: Table S3. This set of 229 proteins were composed of 104 core matrisome proteins (68 ECM glycoproteins, 22 collagen chains and 14 proteoglycans) and 125 ECM-associated proteins (59 ECM regulators, 42 ECM-affiliated proteins and 24 secreted factors). Out of the 229 ECM proteins, 56 proteins were expressed significantly different in IPF lung tissue samples compared with the controls. Among these altered ECM proteins, 19 proteins were upregulated and 37 proteins were downregulated. The distribution of these altered matrisome proteins is presented in Table 1 and Additional file 4: Table S4.

**Table 1:** Numbers of matrisome proteins in lung tissue samples

Matrisome class	Number of ECM proteins	Number of differentially expressed ECM proteins^a^
Collagens	22	11
Glycoproteins	68	15
Proteoglycans	14	1
Affiliated proteins	42	11
Secreted factors	24	6
Regulators	59	12
Total	229	56

^a^More details can be found in Additional files 3 and 4: Tables S3 and S4

### Pathway enrichment analysis of differentially expressed proteins

**Fig. 2:**
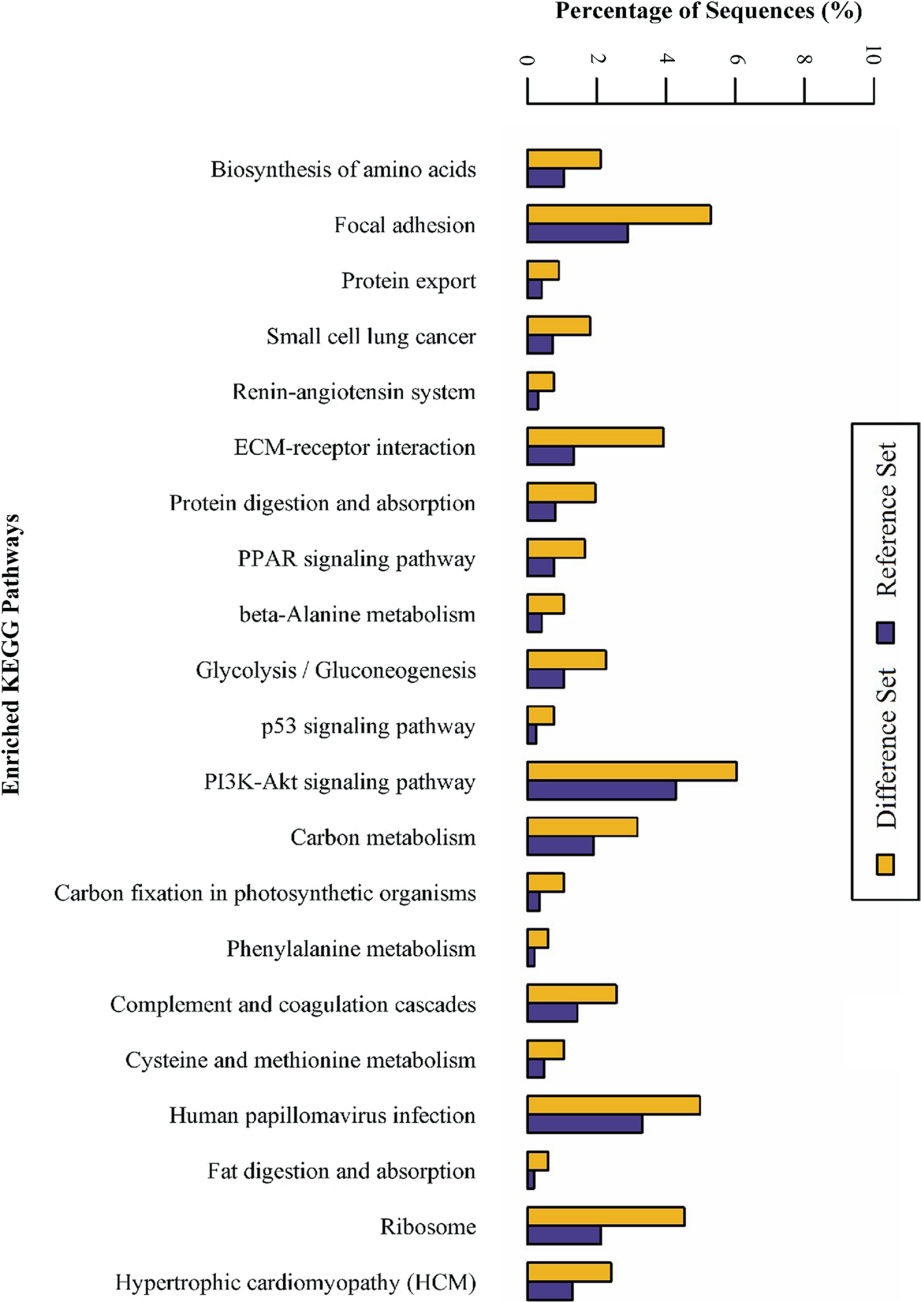
KEGG pathway enrichment analysis of differentially expressed proteins in lung tissue from IPF patients and controls

KEGG pathway enrichment analysis showed that the upregulated proteins in IPF lung tissue mainly belonged to the PI3K-Akt signaling, phagosome, focal adhesion, ECM-receptor interaction, carbon metabolism, human papillomavirus infection and ribosome pathways (Fig. 2 and Additional file 5: Table S5). The PI3 K-Akt signaling pathway was the most representative pathway, encompassing 40 differentially expressed proteins, followed by the focal adhesion and ECM-receptor interaction pathways, encompassing 35 and 26 differentially expressed proteins, respectively.

### Proteins networks analysis

**Fig. 3:**
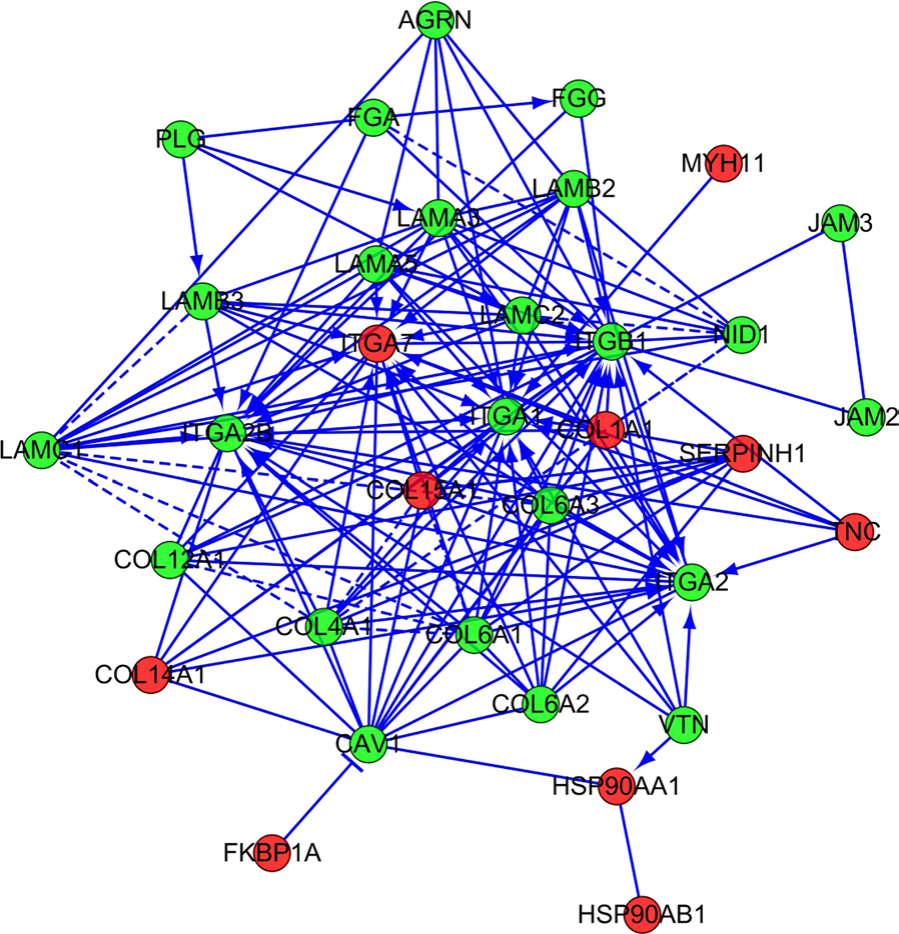
ECM protein interaction network generated using STRING software. Protein–protein interaction regulatory network of differentially expressed ECM proteins between IPF and controls. A dual-color code was used, with red and green indicating up- and down regulation, respectively

IPF is characterized by the redundant deposition and remodeling of the ECM. The KEGG pathway enrichment analysis showed that some of the differentially expressed proteins belonged to the focal adhesion and ECM-receptor interaction pathways. To further our understanding of the regulatory role of ECM in IPF, all disordered ECM proteins were used to build a regulatory network using String software. The result (Fig. 3) demonstrated that these screened differentially expressed ECM proteins formed a complex regulatory network containing 87 nodes and 243 edges with an average node degree of 5.01 and a clustering coefficient of 0.246. The number of expected edges was 12, which was less than the actual 243 edges found. This result indicated that the regulatory ECM network had more interactions than expected, which further indicated that these proteins at least partially biologically connected as a group. COL1A1, COL15A1, ITGA1and ITGA7 were found to be important hubs and were implicated a disorganized ECM protein network in IPF.

**Fig. 4:**
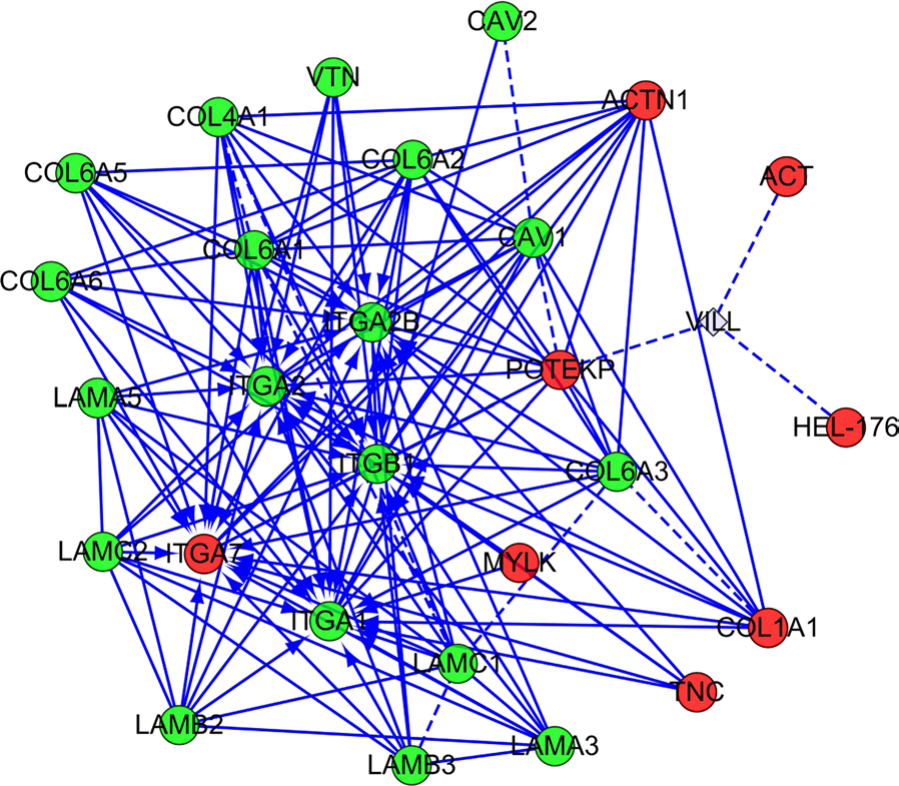
ECM down- and upregulated proteins were collected to build a regulatory network of focal adhesion using STRING software. A dual-color code was used, with red and green indicating up- and down regulation, respectively

Additionally, a regulatory network of focal adhesion proteins in altered ECM proteins were built using String software. The results demonstrated that the differentially expressed ECM proteins formed a complex regulatory network containing 28 nodes and 141 edges with an average node degree of 10.071 and a clustering coefficient of 0.294 (Fig. 4).

### Validation of differentially expressed proteins

**Fig. 5:**
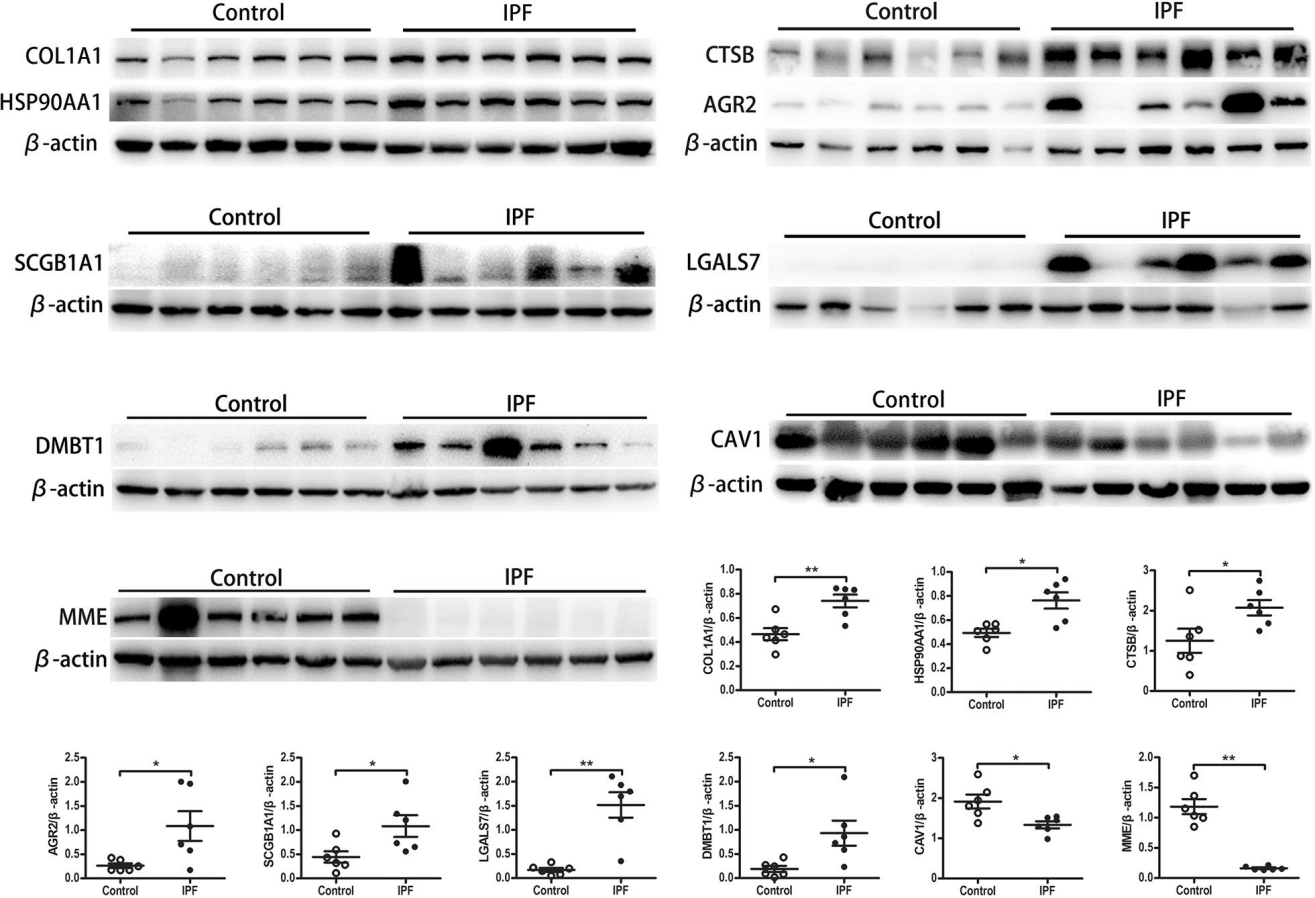
Several altered proteins were selected to verify the results in proteomes analysis by Western blot. Upregulated proteins (COL1A1, CTSB, AGR2, SCGB1A1, HSP90AA1, LGALS7, DMBT1) and two downregulated proteins (MME and CAV1) were detected by Western Blot. Data are shown as the mean ± SEM. *p < 0.05, **p < 0.005. Unpaired, two-tailed Student’s t test

To validate the findings from the proteomics study, several significantly upregulated proteins (COL1A1, CTSB, AGR2, SCGB1A1, HSP90AA1, LGALS7, and DMBT1) and two significantly downregulated proteins (MME and CAV1) were selected for verification in the lung tissue samples by Western blot and immunohistochemistry. As presented in Fig. 5, consistent with the findings in the proteomics study, Western blot revealed that COL1A1, CTSB, AGR2, SCGB1A1, HSP90AA1, LGALS7, and DMBT1 were upregulated, whereas MME and CAV1 were downregulated.

**Fig. 6:**
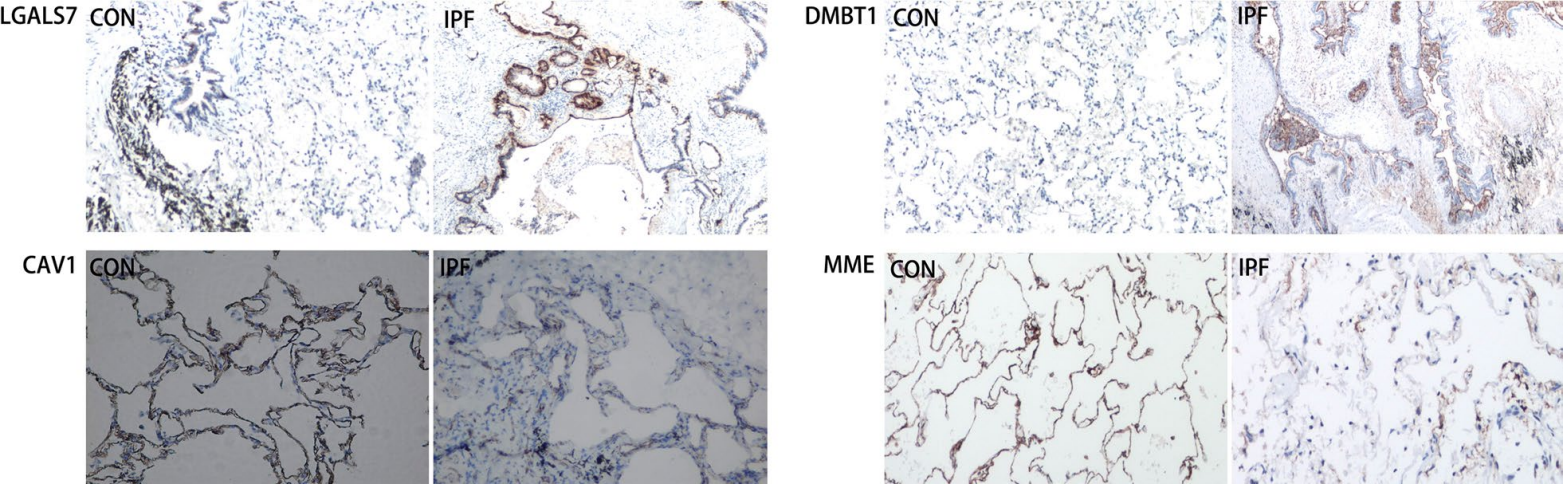
Representative pictures of IHC staining for LGALS7, DMBT1, MME and CAV1 in normal lung tissues and IPF lung tissues (original magnification, 200×)

To identify the cellular sources and locations of differentially expressed proteins in lung tissue, immunohistochemical analyses of LGALS7, DMBT1, MME and CAV1 were performed. Strong positive staining of both LGALS7 and DMBT1 were observed in abnormal bronchiolar structures overlying fibroblast foci and in the hyperplastic bronchioles of IPF lungs, whereas they were much less expressed in the bronchiolar epithelium of control lungs (Fig. 6). As depicted in Fig. 6, consistent with the Western blot results, strong positive expressions of both MME and CAV1 in alveolar epithelial cells were detected in control lungs but were almost absent in IPF lungs.

## Discussion

IPF is a progressive and incurable chronic disease; the discovery of a treatment target is important to IPF patients. A proteomics study of IPF lung tissue is a more accurate way to close the pathogenesis of IPF. A bioinformatics analysis of the proteomics data provides us with many directions to further our study and may help us find a treatment target to prolong the overall survival of IPF patients. However, to the best of our knowledge, relatively limited proteomics studies have focused on IPF lung tissue [23–26]. We applied iTRAQ technology combined with LC-MS/MS to IPF and control lung tissues to obtain proteome profiles of IPF lung tissue. We identified 662 significantly differentially expressed proteins (207 downregulated and 455 upregulated) in IPF lung tissues compared with controls. Briefly, 229 ECM proteins were identified in our proteomics data. Due to the importance of ECM in the pathogenesis of IPF, we studied the characterization of altered ECM proteins in-depth.

To verify the results of the proteomics data, several significantly upregulated proteins previously reported to play roles in the pathology of IPF were chosen [31–39]. Proteins such as SCGB1A1, TAGLN, PSEN2, TSPAN1, CTSB, AGR2, SERPINB3, CSPG2, ASPN [26], HSP90AA1 and HSP90AB1 were included [38]. These proteins are known to be involved in IPF through different mechanisms. For example, SCGB1A1 (CC16, clara cell secretory protein) is a small secreted protein belonging to the secretoglobin family and is a putative anti-inflammatory protein primarily produced by clara cells in the distal airway. Control and IPF lungs showed positive staining in epithelial cells of the airway. Previous studies have also revealed that in addition to clara cells, activated alveolar epithelial cells also contributed to SCGB1A1 production in IPF lung. Compared with non-IPF patients and healthy controls, SCGB1A1 was significantly increased in the serum and BAL fluids of IPF patients, suggesting higher local productions. This finding also indicated that dysfunctional alveolar epithelium played critical roles in aberrant injury/remodeling processes in sporadic and familial IPF [33].

TGF-β is considered one of the most potent inducers of fibroblast activation and pulmonary fibrosis pathogenesis. TGF-β binding to cell surface receptors activates Smad-dependent or Smad-independent pathways. TGF-β induces the translocation of the SMAD2–SMAD3 complex of transcription factors into the nucleus, where it directly promotes the expression of ECM genes, such as COL1A1, COL3A1 and TIMP1, and approximately 60 other ECM-related genes [40]. Consistent with previous researches, we found that some differentially expressed proteins coded by ECM-related genes were regulated by TGF-β. For example, CTSB (cathepsins B) is a member of 11 cysteine cathepsins of the human genome (cathepsins B, H, L, S, C, K, O, F, V, X, and W). Increasing evidence indicate that cysteine cathepsins may be involved in fibrogenesis. The inhibition of murine cathepsins B diminished hepatic inflammation and fibrogenesis [35]. The expression of cathepsins B was increased during the differentiation of stellate cells; these results indicated that cathepsins B participated in liver fibrogenesis [36]. Additionally, the inhibition of cathepsins S may impair the TGF-β1-dependent differentiation of cardiac fibroblasts in a model of myocardial infarction [37]. Kasabova et al. observed that the silencing of cathepsins B or L reduced a-SMA expression; both proteases may be involved in TGF-β1-driven fibroblast differentiation in IPF [34], suggesting that impairment of the cathepsins-dependent proteolytic activity could induce excessive accumulation of collagens and ultimately ECM deposition in the lung. Another protein, TAGLN (transgelin), is a direct target of TGFβ-/Smad3-dependent epithelial cell migration in lung fibrosis [32]. Calabrese et al. reported a strict correlation between TGF-β and squamous cell carcinoma antigen (SCCA) expression in the parenchymal lung tissue of IPF patients [39].

Interestingly, in this study, several proteins of galectins, including galectin-10, galectin-7, galectin-9 and galectin-1, were identified in lung tissues. Among these identified galectins, galectin-7 was significantly increased in IPF patients compared with controls. This was further confirmed by Immunohistochemical staining and Western blot. The galectins belong to a family of beta-galactoside-binding proteins implicated in modulating cell–cell and cell–matrix interactions. These gene products may act as autocrine negative growth factors that regulate cell proliferation. However, many of the effects of galectins are highly tissue specific and context dependent. For example, the genetic deletion of galectin-3 shows different effects in skin compared with lung, heart, and kidney remodeling [41]. Previous studies have reported that galectin-1 and galectin-3 participate in pulmonary fibrogenesis and are mainly regulated by the TGF-β signaling pathway [42, 43]. Multiple cellular functions have been attributed to galectin-7, most of which are related to epithelial integrity maintenance. Galectin-7 has been previously implicated in cell migration during re-epithelialization events after corneal or epidermal injury [44] and in promoting invasiveness during cancer progression [45]. Moreover, galectin-7 has been shown to interfere with transforming growth factor-β (TGF-β) signaling in response to hepatocyte growth factor (HGF) by promoting smad3 export from the nucleus and thus preventing liver fibrosis [46]. Immunohistochemical analysis for identifying cellular localization showed an intense staining for galectin-7 in alveolar and bronchiolar epithelium, alveolar septum and interstitial spaces of IPF lungs. Mild staining was observed in the bronchiolar epithelium of control lungs. These data suggested that increased galectin-7 in lung tissues may be involved in lung remodeling. However, the specific pathophysiological functions of galectin-7 remains to be elucidated in IPF.

Excessive deposition of heterogeneously distributed ECM components in the alveolar parenchyma is a major characteristic of IPF [5, 21]. In addition to the above-mentioned differentially expressed proteins, many ECM components were detected in this study. Recently, the matrisomes of both rodent and human lungs has been identified [24, 29, 30]. While qualitative differences between rodent and human lung ECM were observed, the bulk of matrisome constituents were conserved between the two species. Several studies have addressed ECM compositions in chronic lung diseases such as asthma, COPD and IPF [23–26]. It is comprehensible that different matrisome constituents were indeed identified in these different studies due to the use of different tissue processing methods and ECM protein enrichment methods [27]. For example, although obvious collagen deposition was observed by trichrome staining in IPF lung tissue, the upregulation of collagen type I or III was not detected in acellular human normal and fibrotic lungs by trichrome staining [23, 24]. Conversely, various transcriptome studies of IPF lungs revealed the upregulation of COL1A1 and COL1A2 and of many other collagen genes (e.g., COL3A1, COL5A2, COL6A3, COL14A1, and COL18A1) [12, 13]. Consistent with the above transcriptome studies,22 collagens were identified in our study, and the upregulation of COL1A1 and COL1A2 and other collagens, including COL14A1 and COL15A1 were observed in IPF lungs.

A few studies have focused on the ECM analysis of acellular normal and fibrotic human lung using different detergents, which resulted in the variable loss of proteins, growth factors, and matrix-associated molecules [47]. Previous studies have shown that mild decellularization techniques allow for the near-native retention of laminins, proteoglycans, and other basement membrane and ECM-associated proteins. These near-native retention matrix components bind cells and support biological activity [48]. Recently, Krasny et al. reported that ECM enrichment could lead to systematic increases in core matrisome proteins but result in significant losses of matrisome-associated proteins, including the cathepsins and proteins of the S100 family [49].

Extracellular vesicles (EVs) derived from fibroblast have been identified as an important component of ECM in pulmonary fibrotic disease. Proteomic analysis on EVs extracted from senescent or non-senescent ECM have been performed, the results indicated that the number of EVs in senescent ECM increased significantly compared with non-senescent group. These sequestered EVs mediated fibroblast invasion depending on fibronectin located on its surface [50]. This kind of deep analysis on ECM could increase the possibility to discover therapeutic targets in pulmonary fibrosis.

In our study, we found more ECM proteins than in previous studies [24, 25]. Among the 229 matrisome proteins identified according to the *silico* definition of the matrisome [29, 30], there are 22 collagens, 68 ECM glycoproteins, 59 ECM regulators, 42 ECM-affiliated proteins, 14 proteoglycans and 24 secreted factors. Furthermore, 56 of the 229 ECM proteins were significantly altered, including 11 collagens, 15 ECM glycoproteins, 12 ECM regulators, 11 ECM-affiliated proteins, 1 proteoglycans and 6 secreted factors.

We further compared the ECM components identified in the current study with other IPF related proteomic datasets [23, 26]. For example, Korfei et al. reported that comparative proteomic analyses between IPF lung tissues and donor lung for transplantation, 89 differentially expressed matrisome proteins were identified; 51 out of the 89 were upregulated, and 38 were downregulated in IPF. Among these 89 differentially expressed matrisome proteins, 16 proteins were also found in our identified matrisome proteins. Åhrman et al. established a comprehensive lung tissue proteomics dataset comprising 3621 proteins from an analysis of IPF, COPD and control specimens using a label-free proteomic approach [26]. Consistent with their study, many ECM proteins (e.g., COL1A1, COL14A1, COL15A1, ASPN, COL6A1, COL6A2, SERPINA1SERPINB3,SERPINB9, ADAM9, LAMB1, LAMC1, NID1, ITIH2, CTSB, CTSG and CTSC et al.) were also identified in our ECM proteins.

Not surprisingly, quantitative differences between IPF and control lung tissues were observed in our study. The partial overlap of the ECM components between our study and previous reports supported the feasibility of using iTRAQ technology combined with LC–MS/MS to characterize the ECM composition in lung tissue samples from IPF patients. This method may provide further insights into the molecular mechanisms in pathogenesis of IPF.

## Additional files

**Additional file 1.** The distribution of peptide length.

**Additional file 2.** Differentially expressed proteins.

**Additional file 3.** Identified matrisomal proteins in lung tissue from IPF.

**Additional file 4.** Differentially expressed matrisomal proteins in lung tissues.

**Additional file 5.** KEGG pathway enrichment analysis of differentially expressed proteins.

## References

[1] Raghu G, Collard HR, Egan JJ, Martinez FJ, Behr J (2011). An official ATS/ERS/JRS/ALAT statement: idiopathic pulmonary fibrosis: evidence-based guidelines for diagnosis and management. Am J Respir Crit Care Med.

[2] Travis WD, Costabel U, Hansell DM, King TE, Lynch DA (2013). An official American thoracic society/European respiratory society statement: update of the international multidisciplinary classification of the idiopathic interstitial pneumonias. Am J Respir Crit Care Med.

[3] Raghu G, Rochwerg B, Zhang Y, Garcia CA, Azuma A (2015). An official ATS/ERS/JRS/ALAT clinical practice guideline: treatment of idiopathic pulmonary fibrosis. An update of the 2011 clinical practice guideline. Am J Respir Crit Care Med.

[4] Fernandez IE, Eickelberg O (2012). New cellular and molecular mechanisms of lung injury and fibrosis in idiopathic pulmonary fibrosis. Lancet.

[5] Sgalla G, Iovene B, Calvello M, Ori M, Varone F (2018). Idiopathic pulmonary fibrosis: pathogenesis and management. Respir Res.

[6] Kan M, Shumyatcher M, Himes BE (2017). Using omics approaches to understand pulmonary diseases. Respir Res.

[7] Goodwin AT, Jenkins G (2016). Molecular endotyping of pulmonary fibrosis. Chest.

[8] Hambly N, Shimbori C, Kolb M (2015). Molecular classification of idiopathic pulmonary fibrosis: personalized medicine, genetics and biomarkers. Respirology.

[9] Huang Y, Ma SF, Vij R, Oldham JM, Herazo-Maya J (2015). A functional genomic model for predicting prognosis in idiopathic pulmonary fibrosis. BMC Pulm Med.

[10] Seibold MA, Wise AL, Speer MC, Steele MP, Brown KK (2011). A common MUC5B promoter polymorphism and pulmonary fibrosis. N Engl J Med.

[11] Peljto AL, Zhang Y, Fingerlin TE, Ma SF, Garcia JG (2013). Association between the MUC5B promoter polymorphism and survival in patients with idiopathic pulmonary fibrosis. JAMA.

[12] Konishi K, Gibson KF, Lindell KO, Richards TJ, Zhang Y (2009). Gene expression profiles of acute exacerbations of idiopathic pulmonary fibrosis. Am J Respir Crit Care Med.

[13] Selman M, Pardo A, Barrera L, Estrada A, Watson SR (2006). Gene expression profiles distinguish idiopathic pulmonary fibrosis from hypersensitivity pneumonitis. Am J Respir Crit Care Med.

[14] Yang IV, Burch LH, Steele MP, Savov JD, Hollingsworth JW (2007). Gene expression profiling of familial and sporadic interstitial pneumonia. Am J Respir Crit Care Med.

[15] Herazo-Maya JD, Noth I, Duncan SR, Kim S, Ma SF (2013). Peripheral blood mononuclear cell gene expression profiles predict poor outcome in idiopathic pulmonary fibrosis. Sci Transl Med.

[16] Sun N, Fernandez IE, Wei M, Witting M, Aichler M (2018). Pharmacometabolic response to pirfenidone in pulmonary fibrosis detected by MALDI-FTICR-MSI. Eur Respir J.

[17] Zhang Y, Xin Q, Wu Z, Wang C, Wang Y (2018). Application of isobaric tags for relative and absolute quantification (iTRAQ) coupled with two-dimensional liquid chromatography/tandem mass spectrometry in quantitative proteomic analysis for discovery of serum biomarkers for idiopathic pulmonary fibrosis. Med Sci Monit.

[18] Niu R, Liu Y, Zhang Y, Zhang Y, Wang H (2017). iTRAQ-based proteomics reveals novel biomarkers for idiopathic pulmonary fibrosis. PLoS ONE.

[19] Schiller HB, Mayr CH, Leuschner G, Strunz M, Staab-Weijnitz C (2017). Deep proteome profiling reveals common prevalence of MZB1-positive plasma B cells in human lung and skin fibrosis. Am J Respir Crit Care Med.

[20] Yu G, Ibarra GH, Kaminski N (2018). Fibrosis: lessons from OMICS analyses of the human lung. Matrix Biol.

[21] Knudsen L, Ruppert C, Ochs M (2017). Tissue remodelling in pulmonary fibrosis. Cell Tissue Res.

[22] Herrera J, Henke CA, Bitterman PB (2018). Extracellular matrix as a driver of progressive fibrosis. J Clin Invest.

[23] Korfei M, Schmitt S, Ruppert C, Henneke I, Markart P (2011). Comparative proteomic analysis of lung tissue from patients with idiopathic pulmonary fibrosis (IPF) and lung transplant donor lungs. J Proteome Res.

[24] Booth AJ, Hadley R, Cornett AM, Dreffs AA, Matthes SA (2012). Acellular normal and fibrotic human lung matrices as a culture system for in vitro investigation. Am J Respir Crit Care Med.

[25] Korfei M, Von Der Beck D, Henneke I, Markart P, Ruppert C (2013). Comparative proteome analysis of lung tissue from patients with idiopathic pulmonary fibrosis (IPF), non-specific interstitial pneumonia (NSIP) and organ donors. J Proteom.

[26] Ahrman E, Hallgren O, Malmstrom L, Hedstrom U, Malmstrom A (2018). Quantitative proteomic characterization of the lung extracellular matrix in chronic obstructive pulmonary disease and idiopathic pulmonary fibrosis. J Proteom.

[27] Burgess JK, Mauad T, Tjin G, Karlsson JC, Westergren-Thorsson G (2016). The extracellular matrix—the under-recognized element in lung disease?. J Pathol.

[28] Mullenbrock S, Liu F, Szak S, Hronowski X, Gao B (2018). Systems analysis of transcriptomic and proteomic profiles identifies novel regulation of fibrotic programs by miRNAs in pulmonary fibrosis fibroblasts. Genes (Basel).

[29] Naba A, Clauser KR, Hoersch S, Liu H, Carr SA (2012). The matrisome: in silico definition and in vivo characterization by proteomics of normal and tumor extracellular matrices. Mol Cell Proteom.

[30] Naba A, Clauser KR, Ding H, Whittaker CA, Carr SA (2016). The extracellular matrix: tools and insights for the “omics” era. Matrix Biol.

[31] Tsujino K, Takeda Y, Arai T, Shintani Y, Inagaki R (2012). Tetraspanin CD151 protects against pulmonary fibrosis by maintaining epithelial integrity. Am J Respir Crit Care Med.

[32] Yu H, Konigshoff M, Jayachandran A, Handley D, Seeger W (2008). Transgelin is a direct target of TGF-beta/Smad3-dependent epithelial cell migration in lung fibrosis. Faseb J.

[33] Buendia-Roldan I, Ruiz V, Sierra P, Montes E, Ramirez R (2016). Increased expression of CC16 in patients with idiopathic pulmonary fibrosis. PLoS ONE.

[34] Kasabova M, Joulin-Giet A, Lecaille F, Gilmore BF, Marchand-Adam S (2014). Regulation of TGF-beta1-driven differentiation of human lung fibroblasts: emerging roles of cathepsin B and cystatin C. J Biol Chem.

[35] Canbay A, Guicciardi ME, Higuchi H, Feldstein A, Bronk SF (2003). Cathepsin B inactivation attenuates hepatic injury and fibrosis during cholestasis. J Clin Invest.

[36] Moles A, Tarrats N, Fernandez-Checa JC, Mari M (2009). Cathepsins B and D drive hepatic stellate cell proliferation and promote their fibrogenic potential. Hepatology.

[37] Chen H, Wang J, Xiang MX, Lin Y, He A (2013). Cathepsin S-mediated fibroblast trans-differentiation contributes to left ventricular remodelling after myocardial infarction. Cardiovasc Res.

[38] Bellaye PS, Shimbori C, Yanagihara T, Carlson DA (2018). Synergistic role of HSP90alpha and HSP90beta to promote myofibroblast persistence in lung fibrosis. Eur Respir J.

[39] Calabrese F, Lunardi F, Giacometti C, Marulli G, Gnoato M (2008). Overexpression of squamous cell carcinoma antigen in idiopathic pulmonary fibrosis: clinicopathological correlations. Thorax.

[40] Verrecchia F, Chu ML, Mauviel A (2001). Identification of novel TGF-beta/Smad gene targets in dermal fibroblasts using a combined cDNA microarray/promoter transactivation approach. J Biol Chem.

[41] Mcleod K, Walker JT, Hamilton DW (2018). Galectin-3 regulation of wound healing and fibrotic processes: insights for chronic skin wound therapeutics. J Cell Commun Signal.

[42] Kathiriya JJ, Nakra N, Nixon J, Patel PS, Vaghasiya V (2017). Galectin-1 inhibition attenuates profibrotic signaling in hypoxia-induced pulmonary fibrosis. Cell Death Discov.

[43] Mackinnon AC, Gibbons MA, Farnworth SL, Leffler H, Nilsson UJ (2012). Regulation of transforming growth factor-beta1-driven lung fibrosis by galectin-3. Am J Respir Crit Care Med.

[44] Gendronneau G, Sidhu SS, Delacour D, Dang T, Calonne C (2008). Galectin-7 in the control of epidermal homeostasis after injury. Mol Biol Cell.

[45] Advedissian T, Deshayes F, Viguier M (2017). Galectin-7 in epithelial homeostasis and carcinomas. Int J Mol Sci.

[46] Inagaki Y, Higashi K, Kushida M, Hong YY, Nakao S (2008). Hepatocyte growth factor suppresses profibrogenic signal transduction via nuclear export of Smad3 with galectin-7. Gastroenterology.

[47] Calle EA, Hill RC, Leiby KL, Le AV, Gard AL (2016). Targeted proteomics effectively quantifies differences between native lung and detergent-decellularized lung extracellular matrices. Acta Biomater.

[48] Krasny L, Bland P, Kogata N, Wai P, Howard BA (2018). SWATH mass spectrometry as a tool for quantitative profiling of the matrisome. J Proteom.

[49] Krasny L, Paul A, Wai P, Howard BA, Natrajan RC (2016). Comparative proteomic assessment of matrisome enrichment methodologies. Biochem J.

[50] Chanda D, Otoupalova E, Hough KP, Locy ML, Bernard K (2018). Fibronectin on the surface of extracellular vesicles mediates fibroblast invasion. Am J Respir Cell Mol Biol..

